# Comparison of LC-MS/MS and EMIT methods for the precise determination of blood sirolimus in children with vascular anomalies

**DOI:** 10.3389/fphar.2022.925018

**Published:** 2022-09-06

**Authors:** Yue-Tao Zhao, Hao-Ran Dai, Yue Li, Yuan-Yuan Zhang, Hong-Li Guo, Xuan-Sheng Ding, Ya-Hui Hu, Feng Chen

**Affiliations:** ^1^ Pharmaceutical Sciences Research Center, Department of Pharmacy, Children’s Hospital of Nanjing Medical University, Nanjing, China; ^2^ School of Basic Medicine and Clinical Pharmacy, China Pharmaceutical University, Nanjing, China

**Keywords:** sirolimus, EMIT, LC-MS/MS, TDM, consistency, children, vascular anomalies

## Abstract

Sirolimus (SRL) is a mammalian target of rapamycin (mTOR) inhibitor. The whole blood concentration of SRL is routinely monitored to tailor dosage and prevent toxicity. Currently, the enzyme multiplied immunoassay technique (EMIT) is often applied to perform therapeutic drug monitoring (TDM) of SRL, but the cross-reactivity with various metabolites is of great concern. A more specific method is required, such as liquid chromatography–tandem mass spectrometry (LC-MS/MS). However, no study on the method comparison of the EMIT and LC-MS/MS for the measurement of whole blood SRL concentration in children with vascular anomalies has been reported. This study developed a simple and sensitive LC-MS/MS assay for the determination of SRL. Meanwhile, consistency between LC-MS/MS and the EMIT was evaluated by linear regression and Bland–Altman analysis. Whole blood samples were deproteinized with methanol for erythrocyte lysis, and the resulting solution was injected into the LC-MS/MS system using the positive electrospray ionization mode. The multiple reaction monitoring transitions of *m/z* 931.7 → 864.6 and *m/z* 934.7 → 864.6 were used for SRL and SRL-d_3_ as the internal standards, respectively. The analytes were separated on a C18 column with a gradient mobile phase (0.1 mM formic acid and 0.05 mM ammonium acetate in methanol/ultrapure water). Blood samples collected from children with vascular anomalies undergoing SRL therapy were tested by EMIT and by LC-MS/MS. The linear range of LC-MS/MS was 0.500–50.0 ng/ml and that of the EMIT was 3.50–30.0 ng/ml. A significant positive correlation between the two assays was established with a regression equation described as [***EMIT***] = 1.281 × [***LC−MS/MS***] + 2.450 (*r* = 0.8361). Bland–Altman plots showed a mean concentration overestimation of 4.7 ng/ml [95% CI: (−3.1, 12.6)] and a positive bias of 63.1% [95% CI: (−36.1, 162.3)] generated by the EMIT more than that of by LC-MS/MS. In conclusion, the two methods were closely correlated, indicating that switching between the two methods is feasible. Considering the overestimation nature of the EMIT assay, switching from the EMIT to the LC-MS/MS method deserves close attention and necessary re-evaluation for the target therapeutic reference range, may be required when methods are switched within the same clinical laboratory or results are compared between different laboratories.

## 1 Introduction

Sirolimus (SRL) is a hydrophobic macrolide compound which was first isolated and developed as an antifungal drug (Sehgal, Baker et al., 1975; Vezina, Kudelski et al., 1975). Simultaneously, SRL exerts intensively immunosuppressive and antiproliferative activities due to its ability to inhibit the mammalian target of rapamycin (mTOR) (Sehgal 1995; Laplante and Sabatini, 2012). However, SRL has a narrow therapeutic window, and its clinical pharmacokinetics exhibits large intra- and inter-patient variability (Kahan, Napoli et al., 2000; Scott, Courter et al., 2013). Its side effects correlate closely to whole blood concentration; thus, the implementation of therapeutic drug monitoring (TDM) in whole blood samples for SRL is essential and beneficial to individualize dose regimens and ensure its efficacy and safety (Vogeser, Fleischer et al., 2002; Mano, Sato et al., 2011).

To routinely monitor the blood level of SRL, immunoassay methods have traditionally been involved (Sallustio, Noll et al., 2011). The enzyme multiplied immunoassay technique (EMIT) has been widely used for assaying endogenous and exogenous substances for a long time, and it is particularly useful in clinical TDM (Borgman, Hiemer et al., 2012). However, the EMIT shows poor specificity as it cannot distinguish the target analyte from its metabolite(s), which causes positive bias from true concentration values. Impressively, in our laboratory, where the EMIT assay has been applied to detect the whole blood SRL concentration for nearly 5 years for children with vascular anomalies, this method is still accompanied by some other weaknesses, including relatively high fluctuation of quality control (QC) samples and expensive reagent kit expenditure with a short validity period. Hence, more specific assays with better sensitivity and selectivity are required to alternatively measure the whole blood SRL concentration.

Liquid chromatography–tandem mass spectrometry (LC-MS/MS) has been widely applied for the analysis of low molecular weight molecules with the strengths of low interference, good selectivity, high degree of sensitivity, high throughput, and low costs per sample in terms of reagents (van den Ouweland and Kema, 2012). It allows the accurate determination of the target analyte(s) and/or its metabolites and ensures reliable results and superiority over other assays such as the EMIT (Nguyen, Duong et al., 2021). In clinical laboratories, the LC-MS/MS instrumentation provides great accuracy and is very suitable for routine TDM (Shipkova and Svinarov, 2016; Cui, Wang et al., 2020).

Recently, several LC-MS/MS methods with time-consuming solid-phase extraction, large sample size requirements or longer run time for each individual sample have been reported ((Salm, Taylor et al., 2000; Zochowska, Bartlomiejczyk et al., 2006; O'Halloran and Ilett, 2008; Morgan, Brown et al., 2014, Shi et al., 2016); Table 1). Some assays were suitable for routine TDM, but some others were not. Hence, the aims of this study were 1) to develop and validate an easy-to-use LC-MS/MS method for the analysis of SRL whole blood concentration; 2) to assess the method consistency between the newly validated LC-MS/MS and routine EMIT technique for SRL determination in our laboratory; and 3) to discuss the feasibility and necessity of the method switching from the EMIT to LC-MS/MS for routine SRL monitoring in clinical laboratories.

**TABLE 1:** Comparison of this study with several previously published analytical methods for SRL.

Study	Method	Internal standard	Blood volume (μl)	Sample preparation	Elution	Column	Mobile phase	Linearity range (ng/ml)	Analytical time (min)	Accuracy (%)
Salm, Taylor et al. (2000)	HPLC-MS	32-O-desmethoxysirolimus	500	PPT by ACN and ZnSO_4_, followed by SPE	Isocratic	Novapak C18 column (150 mm × 2.1 mm, 4 μm)	80% MeOH, 20% 50 mM NH_4_AC, pH 5.1	0.2–100	10	94.4–104.4
Zochowska, Bartlomiejczyk et al. (2006)	HPLC-UV	Desmethoxyrapamycin	1500	PPT and extracted with 1-chlorobutan	Isocratic	Supelco RP C16-Amide column (150 mm × 4.6 mm, 5 μm)	60% ACN in water	3–50	15	NR
O'Halloran and Ilett (2008)	LC-MS/MS	Desmethoxyrapamycin Sirolimus-d_3_	15	PPT by ACN and ZnSO_4_	Gradient	Supelco C18 column (250 mm × 4.6 mm; 5 µm)	1 ml/L FA and 2 mM NH_4_AC in MeOH and water	1–50	2.5	NR
Morgan, Brown et al. (2014)	LC-MS/MS	^13^C_2_D_4_-everolimus	20	PPT by ACN and NH_4_HCO_3_ and ZnSO_4_	Gradient	Waters Symmetry C18 column (50 mm × 2.1 mm, 3.5 μm)	2 mM NH_4_AC and 0.1% FA in MeOH and water	1–49	6	90.7–113.16
Shi et al. (2016)	PS-MS/MS	Sirolimus-d_3_	200	PPT by MeOH and dried	NR	NR	NR	LLOQ: 2	NR	NR
This study	LC-MS/MS	Sirolimus-d_3_	100	PPT by MeOH	Gradient	Kinetex C18 column (50 mm × 2.1 mm, 1.7 μm)	0.1 mM FA and 0.05 mM NH_4_AC in MeOH and water	0.5–50	3	88.7–111.8

Abbreviations: HPLC-MS, high-performance liquid chromatography–mass spectrometry; HPLC-UV, high-performance liquid chromatography–ultraviolet; LC-MS/MS, high-performance liquid chromatography–tandem mass spectrometry; PS-MS/MS, paper spray–tandem mass spectrometry; PPT, protein precipitation; ACN, acetonitrile; ZnSO_4_, zinc sulfate; NH_4_HCO_3_, ammonium bicarbonate; MeOH: methanol; SPE, solid-phase extraction; NR, not reported; C18, octadecyl carbon chain; NH_4_AC, ammonium acetate; FA, formic acid; LLOQ, lower limit of quantitation.

## 2 Materials and methods

### 2.1 Liquid chromatography–tandem mass spectrometry method

#### 2.1.1 Chemicals, reagents, and materials

The reference material of SRL (purity: 95%, Lot No. 8-RTU-49-1, expiry date: 2023-04-09) and its isotopically labeled internal standard (IS), SRL-d_3_ (technical grade, Lot No. 3-TKA-137-3, expiry date: 2023-10-08) were purchased from the Toronto Research Chemicals Inc. (Toronto, Canada). HPLC-grade methanol (MeOH) was bought from Merck KGaA (Darmstadt, Germany). ACS-grade formic acid (FA) and ammonium acetate (NH_4_AC) were obtained from Tedia Company Inc. (Fairfield, OH, United States) and Sigma-Aldrich Co. LLC (Wilmington, United States), respectively. Ultrapure water (UPW) was generated from a Milli-Q water purification system (Millipore Corp., Bedford, MA, United States).

Chromatographic columns including Kinetex 1.7 μm C18 100 Å (50 mm × 2.1 mm), Luna 5 μm C18 100 Å (50 mm × 2.0 mm), Gemini 3 μm C18 110 Å (50 mm × 2.0 mm), Kinetex 2.6 μm C18 100 Å (50 mm × 2.1 mm), Kinetex 5 μm C18 100 Å (50 mm × 2.1 mm), and security guard cartridges C18 (4 mm × 2.0 mm) were purchased from the Phenomenex Inc. (Torrance, CA, United States).

Cryopreserved human whole blood samples were supplied by the therapeutic drug monitoring lab (Children’s Hospital of Nanjing Medical University, Nanjing, China), which were left-over samples from the clinical testing. The study was performed in accordance with the Helsinki Declaration, and the study protocol was approved by the Children’s Hospital of Nanjing Medical University ethics committee (protocol number 202206114-1). This study aimed to evaluate the analytical consistency of the whole blood SRL levels generated by an EMIT assay and by an LC-MS/MS method, but no clinical and personal data were reported. Thus, the consent to participate is not applicable.

#### 2.1.2 Liquid chromatography–tandem mass spectrometry conditions

This separation method was developed on a Jasper™ liquid chromatography system (AB Sciex Pte. Ltd., Singapore) with a binary pump (Sciex Dx™), an online degasser (Sciex Dx™), an auto-sampler (Sciex Dx™), and a column oven (Sciex Dx™). Liquid chromatographic (LC) separation was performed on a Kinetex C18 column, protected by a security guard C18 cartridge.

The mobile phase consisted of UPW (phase A) and MeOH (phase B), both containing 0.1 mM FA and 0.05 mM NH_4_AC. A gradient elution with a flow rate at 0.4 ml/min was programmed as follows: 0–0.4 min, 50% phase B; 0.4–0.41 min, 50–90% phase B; 0.41–0.85 min, 90–100% phase B; 0.85–1.8 min, 100% phase B; 1.8–2.2 min, 100–50% phase B; 2.2–3.0 min, 50% phase B. The analytical run time was 3.0 min, and the LC flow was only directed into the MS between 1.0 and 3.0 min. The temperature for the column and auto-sampler was 50°C and 4°C, respectively.

MS detection of SRL and SRL-d_3_ was conducted using a Triple Quad™ 4500MD system (AB Sciex Pte. Ltd., Singapore), equipped with an electrospray ionization (ESI) source. Quantification was operated in the positive ESI mode [ESI (+)], with multiple reaction monitoring (MRM) as the acquisition mode. The transitions and conditions are shown in Table 2. Other settings are as follows: curtain gas (CUR): 25 psi; collision-activated dissociation (CAD): 6 units; ion spray voltage: 5500 V; nebulizer gas (GS1): 40 psi; heater gas (GS2): 40 psi; ion source house temperature (TEM): 550°C. The LC-MS/MS system control and data analysis were performed using Analyst MD software (Version 1.6.3, AB Sciex Pte. Ltd., Singapore).

**TABLE 2:** MRM transitions and conditions of SRL and SRL-d_3_.

Compound	Transitions (*m/z*)	DP (V)	EP (V)	CE (V)	CXP (V)
SRL	931.7 → 864.6	24.0	5.00	23.0	21.0
SRL-d_3_	934.7 → 864.6	17.0	8.00	26.0	30.0

Abbreviations: DP, declustering potential; EP, entrance potential; CE, collision energy; CXP, collision cell exit potential.

#### 2.1.3 Preparation of solutions, calibration standards, and quality control samples

Stock solutions of SRL (1.00 mg/ml) and SRL-d_3_ (1.00 mg/ml) were dissolved in MeOH. SRL stock solutions were further diluted with MeOH: H_2_O (1:1; v/v) to prepare working solutions. All the stock solutions and working solutions were stored at −80°C before use.

Calibration standards and QC samples were prepared by spiking the working solutions into a blank matrix (human whole blood) at a ratio of 1: 20 to achieve serial concentrations of calibration standard samples. The calibration curve was prepared at 0.500, 1.00, 2.00, 5.00, 10.0, 30.0, and 50.0 ng/ml. The following QC samples with concentration levels were 0.500 ng/ml (lower limit of quantification QC, LLOQ QC), 1.50 ng/ml (low QC, LQC), 15.0 ng/ml (medium QC, MQC), and 40.0 ng/ml (high QC, HQC).

#### 2.1.4 Sample preparation

The aliquot of 100 μl of the whole blood sample was pipetted into a 1.5-ml Eppendorf tube. An aliquot of 200 μl of MeOH containing IS (15 ng/ml of SRL-d_3_) was added, followed by 300 μl of neat MeOH solvent. The mixture was vortexed for 10 min. After centrifugation at 12,000 rpm for 10 min at 4°C, 10 μl of the supernatant extract was injected into the LC-MS/MS system for analysis.

### 2.2 Method validation

The present method was optimized and validated using cryopreserved and fresh whole blood as matrices according to the Bioanalytical Method Validation Guidance for Industry published by the U.S. Food and Drug Administration (FDA) in 2018 (U.S. Food and Drug Administration, 2018).

#### 2.2.1 Selectivity

Double blank samples from six different individual sources of the matrix were used to evaluate the selectivity of the analysis. The interference between the analyte and IS was assessed using human whole blood samples of zero blank containing IS and the upper limit of quantification (ULOQ) without IS.

#### 2.2.2 Linearity and lower limit of quantification

Linearity of the LC-MS/MS assay was tested by analysis of all calibrators that were run in duplicate at the beginning and end of each batch, with concentrations ranging from 0.500 to 50.0 ng/ml for SRL. The ratio of the standard peak area to the IS peak area was plotted against the ratio of the standard concentration/IS for constructing calibration curves, and a 1/x^2^ weighting factor was used for linear regression.

The LLOQ, defined as the lowest point of the calibration curve, should be within an acceptable range for method accuracy and precision, and the signal-to-noise ratio (S/N) should be no less than 5.

#### 2.2.3 Accuracy and precision

Four concentration levels of QC samples (LLOQ QC, LQC, MQC, and HQC) in six replicates were assessed for determination of the intra-batch accuracy and precision. The inter-batch accuracy and precision were established by the repeat of the intra-batch validation procedure in three consecutive batches prepared on different days. Accuracy is expressed as a relative error (RE, %), and precision is expressed as the relative standard deviation (RSD, %).

#### 2.2.4 Recovery and matrix effect

Recovery was tested by spiking equal amounts of SRL and IS into aliquots of blank whole blood before and after extraction. The experiment was performed using three concentration levels (LQC, MQC, and HQC) for SRL, and each was measured six times. The recovery was calculated from the signal intensity ratios of the samples spiked before preparation to the samples spiked after preparation. The matrix effect was evaluated using six different sets of extracted blank blood samples and methanol samples with equal volumes of the analyte and IS added by repeated measurements (*n* = 3). To determine the matrix effect, the mean peak area of the blank blood samples that were extracted and spiked with the analyte and IS at the designated concentration was compared to the mean peak area of matrix-free, methanol-enriched samples. The matrix effects of SRL and IS were calculated in the same way, and then the matrix effect was assessed by IS-normalized matrix factors.

#### 2.2.5 Stability

The stability of the analyte in the matrix was determined by LQC and HQC samples in triplicates after being kept in various storage conditions: room temperature, −80°C, and five freeze–thaw cycles. The post-preparative stability was tested by reanalyzing the LQC and HQC samples stored in the auto-sampler (4°C).

### 2.3 Enzyme multiplied immunoassay technique assay

According to the package insert, the Emit® 2000 SRL Assay is a homogeneous enzyme immunoassay containing mouse monoclonal antibodies with a high specificity for SRL. This EMIT assay is based on a competition of SRL antibody binding sites. SRL in the sample competes with SRL in the enzyme reagent that is labeled with recombinant enzyme glucose 6-phosphate dehydrogenase (rG6PDH). Active (unbound) rG6PDH enzyme converts the oxidized nicotinamide adenine dinucleotide (NAD) in the antibody reagent to NADH, resulting in a kinetic absorbance change that can be measured spectrophotometrically. Enzyme activity decreases upon binding to the antibody, allowing SRL concentrations to be measured in terms of enzyme activity. The liquid assay reagent kit (Siemens Healthcare Diagnostics Inc., Newark, NJ, United States) contains antibody reagent 1, buffer reagent 2, and enzyme reagent 3.

#### 2.3.1 Reagents

Emit® 2000 Sirolimus Calibrators (Lot No. P1; expiry date: 2022-03-02), Emit® 2000 Sirolimus Assay (Lot No. P1; expiry date: 2022-04-09), and Emit® 2000 Sirolimus Sample Pretreatment Reagent (Lot No. N2; expiry date: 2023-05-10) were obtained from the Siemens Healthcare Diagnostic Ltd. (Newark, NJ, United States). Controls of SRL (Lot No. 0336; expiry date: 2024-11-30) were supplied by Bio-Rad Laboratories Inc. (Irvine, United States).

#### 2.3.2 Assay performance

Sample pretreatment is required for red blood cell lysis, SRL solubilization, and protein precipitation prior to measurement on the EMIT analyzer. This was accomplished by adding 50 μl of the sample pretreatment reagent (Siemens Healthcare Diagnostics Inc.) and 200 μl of MeOH to 200 μl of real whole blood samples, calibrators, or controls in micro-centrifuge tubes. The samples were then vortexed for 5 min, followed by standing at room temperature for another 2 min, and then centrifuged at 12,000 rpm for 5 min at 4°C. The resulting supernatant is decanted and measured on the analyzer.

The SRL assay was carried out for 20 min. The following instrument parameters were established: a pretreated sample (28 μl) was added to reagent 1 (120 μl) and reagent 2 (60 μl). Following a 130-s incubation at 37°C, reagent 3 (60 μl) was added. The reaction mixture was monitored at 340 nm, 106 s after the addition of reagent 3. Using SRL calibrators analyzed in duplicate, the data were fitted to a parametric logit mathematical equation. Sample results were calculated by the instrument from the stored calibration curve.

The whole blood SRL concentration was assayed using an automated enzyme immunoassay analyzer (SIEMENS, Munich, Germany). The calibration curve of the assay was prepared at 0.00, 3.00, 6.00, 12.0, 24.0, and 36.0 ng/ml. QC samples were accepted if the deviation did not exceed ±15% to ensure the accuracy and precision of the EMIT method.

### 2.4 Comparison of sirolimus concentrations generated by liquid chromatography–tandem mass spectrometry and by the enzyme multiplied immunoassay technique

Post completing the routine SRL monitoring by the EMIT assay and reporting results to clinicians, the left-over whole blood specimens were determined again by the newly validated LC-MS/MS. All samples were collected between June and December 2021. These samples are routinely transported to our laboratory for monitoring the whole blood SRL levels in children with vascular anomalies. In brief, 114 blood samples were collected from 49 children at the Department of Orthopedics, Children’s Hospital of Nanjing Medical University. The concentration results generated by LC-MS/MS and the EMIT were compared statistically.

### 2.5 Morphological examination of red blood cells

Blood smears were stained with Wright–Giemsa stain (Baso Diagnostics Inc., Zhuhai City, Guangdong Province, China). First, an aliquot of 0.5–0.8 ml solution A was dropped onto smears and stained for 1 min. Then, solution B was added to solution A (the volume of solution B was two to three times that of solution A). A ear washing bulb was used to make the liquid surface ripple by blowing out the breeze and mixing two solutions thoroughly. After staining for 4–10 min, the smears were rinsed. Dried smears were examined under a BX51 microscope (Olympus Corp., Tokyo, Japan), and images were collected through J D 801 series medical imaging workstation system software.

### 2.6 Statistical analysis

GraphPad Prism Software (version 8.3.0, CA, United States), Medcalc software (Ostend, Belgium), and Analyse-it Software (version 5.66, Leeds, United Kingdom) were used to statistically analyze all data. Linear regression analysis was performed by GraphPad to estimate the association between the assays. The Bland–Altman difference plot, which can be drawn by MedCalc software, is helpful in demonstrating the potential relationship between the differences and the magnitude of measurements exhibiting any systematic bias and in identifying possible outliers. Weighted Deming regression was performed by Analyse-it to complete the data comparison.

## 3 Results and Discussion

### 3.1 Optimization of the analytical method

#### 3.1.1 Mass spectrometric parameter optimization

**FIGURE 1:**
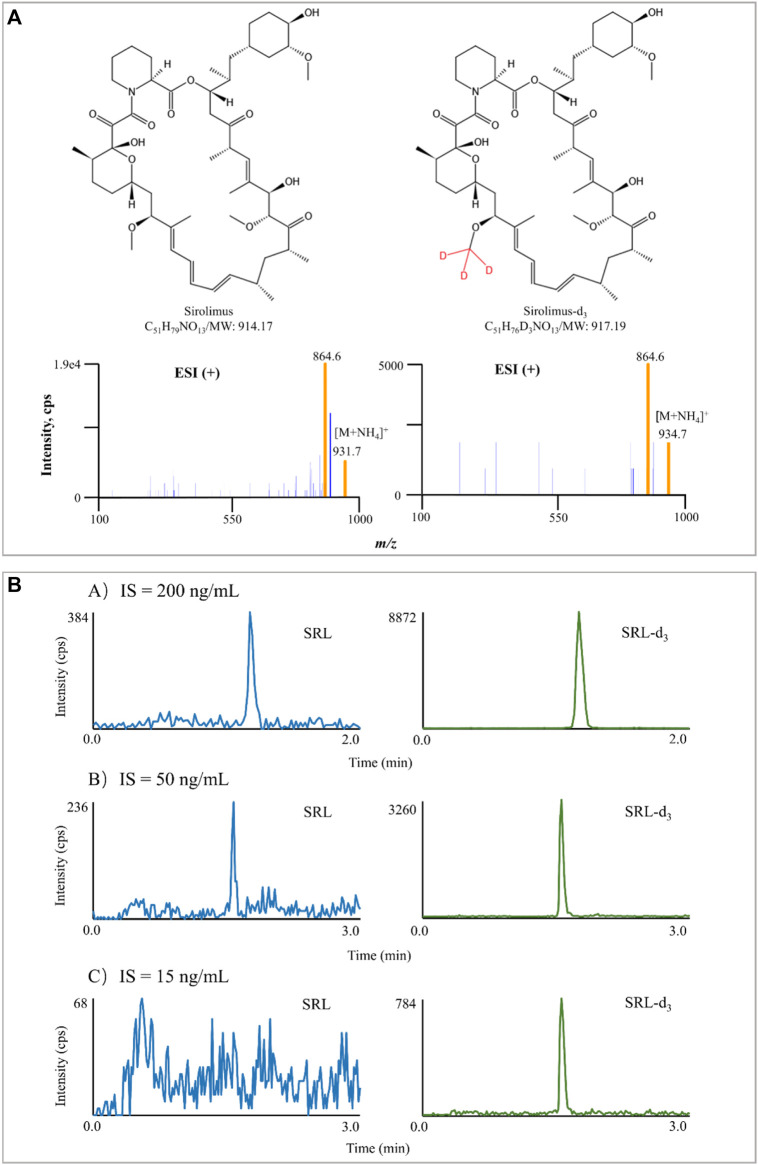
Typical MS/MS product ion spectra of SRL and SRL-d_3_. The experiment was performed under *Manual Tune* mode by a syringe infusing the standard solution of SRL and SRL-d_3_ (100 ng/ml) at a rate of 5 μl/min **(A)**. Interference of IS in blank samples spiked with IS only. The difference in chromatographic run time in **(A)** is due to the different gradient **(B)**.

The majority of LC-MS/MS methods for measurement of SRL concentration have used ESI as the ion source (Koster, Dijkers et al., 2009; Ivanova, Artusi et al., 2011; Koster, Alffenaar et al., 2013; Yuan, Payto et al., 2014; Morgan, Nwafor et al., 2016). SRL did not readily protonate under ESI conditions because it is neutral, but SRL will preferentially form adducts with cations (e.g., Na^+^, K^+^, and NH_4_^+^) (Taylor 2004). Bogusz, Enazi et al. (2007) revealed the presence of the ammoniated adduct of SRL through syringe infusion experiments in the mobile phase in full scan mode. In line with Bogusz, Enazi et al. (2007), the ammonium adduct ions were confirmed for SRL and SRL-d_3_ in this study. The MS/MS spectra of SRL and SRL-d_3_ are shown in Figure 1A.

#### 3.1.2 Mobile phase and gradient elution optimization

During initial method development, an attempt was made to optimize the mobile phase. Mobile phase selection is critical since it affects analyte selectivity and resolution. At first, MeOH was chosen as the organic phase because it was commonly used in our laboratory and was relatively economic and less toxic. No improvement in signal intensity or peak shape was found when ACN was alternatively tested as the mobile phase B. Thus, the mobile phase consisted of UPW (phase A) and MeOH (phase B). In addition to the properties of the target analyte and mobile phase composition, the solution environment is also critical for the sensitivity of ESI-based MS detection because of its key role in the nebulization and ionization process. The mobile phase modifiers (including the type and percentage of the organic solvent used and the type and concentration of the electrolyte added) affect the ionization efficiency and MS response of the target analytes (Li, Tian et al., 2012). In the current study, FA and NH_4_AC were next examined for the candidate modifiers. Seven concentration levels of FA (0.008, 0.04, 0.1, 0.2, 0.5, 1, and 5 mM) were tested, and the optimal result appeared to be achieved with the 0.1 mM FA-modified mobile phase. Due to the [M + NH_4_]^+^ as the MRM transitions, eight concentration levels of NH_4_AC (0.05, 0.1, 0.2, 0.5, 1, 2, 5, and 10 mM) were further examined, and the best result seemed to be achieved under the 0.05 mM NH_4_AC. Collectively, the mobile phase contained 0.1 mM FA and 0.05 mM NH_4_AC.

In the reversed-phase LC, method development often starts with a gradient elution separation. From such separation, it is likely to evaluate whether isocratic or gradient elution is appropriate for a given target analyte or more and test either the solvent strength for isocratic separations or the gradient range for gradient elution. Gradient elution is more attractive as it offers a favorable approach to significantly reduce the time required (Jandera, 2006). In this study, we found that SRL and SRL-d_3_ were efficiently eluted at a high proportion of MeOH during the early stages of the experiments. The starting proportion of the UPW phase was 50%, and the MeOH percentage in the equivalent elution was examined from 80% to 100%. At last, the optimal elution ability and ionization efficiency were acquired when the organic phase proportion was 100%.

#### 3.1.3 Selection of the chromatographic column

The particle size of a column packing affects the efficiency (theoretical plates) of a column. Smaller particle size helps optimize the performance of the LC-MS/MS method, attributing to shorter column lengths, higher optimum eluent velocities, and lower theoretical plate heights (Chen, Li et al., 2014). In addition, smaller particles can be used to enhance chromatographic resolution and decrease the analysis time (Nguyen, Guillarme et al., 2006). Therefore, in this study, we next investigated the influence of different columns on the performances of SRL and SRL-d_3_ when other chromatographic conditions remained unchanged. The particle size varied from 1.7 to 5 μm, while the column length was fixed at 50 mm. Finally, the C18 column with a 50-mm length (2.1 mm ID) and 1.7-μm particle size in diameter (pore size, 100 Å) was selected, thereby achieving the optimal response and peak shape.

#### 3.1.4 Internal standard concentration selection

In addition to the mobile phase used, chromatographic separation, and sample processing, the IS selection also contributes to the method performance (Valbuena, Shipkova et al., 2016). The IS compensates for those unavoidable assay variances and is widely used in quantitative LC-MS/MS bioanalysis for improving both the precision and accuracy of the assay (Lowes, Jersey et al., 2011). Ideally, a stable-isotope labeled chemical is preferred as it has exactly the same structure as the analyte and co-elutes with it (Fu, Barkley et al., 2020). In this study, SRL-d_3_ was utilized, and the concentration of SRL-d_3_ was initially set at 200 ng/ml, but the IS interfered seriously with SRL there. The SRL-d_3_ concentration was subsequently reduced to 50 ng/ml, but the interference still existed. Interestingly, when the SRL-d_3_ level was set at 15 ng/ml, the interference could be ignored and the MS response of the IS became stable (Figure 1B).

### 3.2 Sample cleanup optimization

**FIGURE 2:**
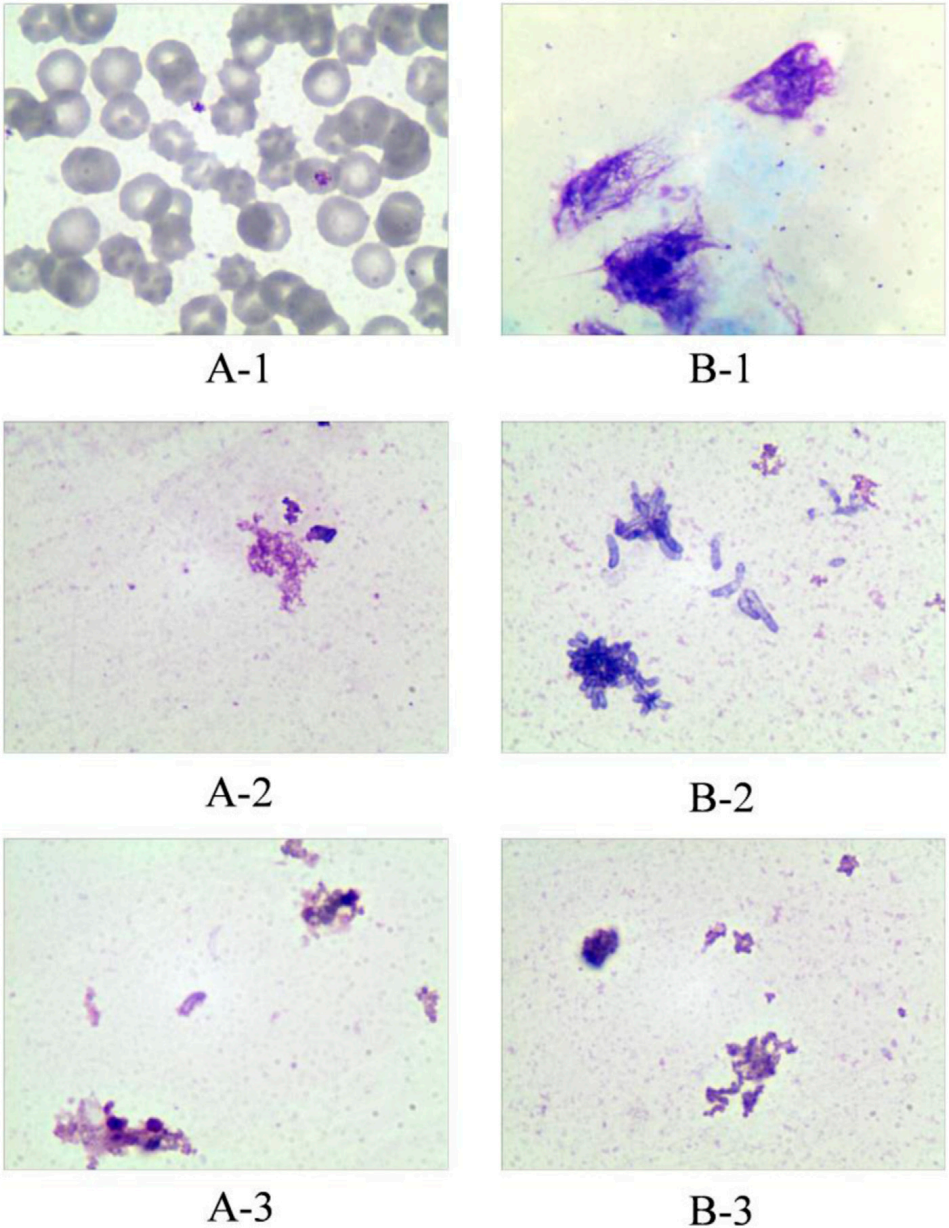
Effect of different treatments on red blood cell disruption. Group A represents fresh whole blood; group B represents cryopreserved whole blood. A-1: Fresh whole blood spiked only; A-2: fresh whole blood precipitated with methanol and mixed up-and-down; A-3: fresh whole blood precipitated with methanol and vortexed for 10 min. B-1: Cryopreserved whole blood spiked only; B-2: cryopreserved whole blood precipitated with methanol and mixed up-and-down; B-3: cryopreserved whole blood precipitated with methanol and vortexed for 10 min.

SRL was distributed predominantly (about 95%) into red blood cells (RBCs), with only a small proportion of the drug being found in the plasma fraction (Stenton, Partovi et al., 2005). From the TDM standpoint, the preferred matrix for SRL measurement would be whole blood (Yatscoff, LeGatt et al., 1993). Optimum recovery of SRL from whole blood has proven to be problematic such as crosstalk interference and sacrificed recovery due to inappropriate clean-up methods in the past when compared with other common immunosuppressant drugs (Morgan, Brown et al., 2014). Therefore, the ability to lyse RBCs of the cleanup method can affect the results of concentration measurement. Therefore, in this study, we investigated the efficiency of the sample processing method on RBC lysis. Cryopreserved whole blood and fresh whole blood were treated in different ways and then stained with Wright–Giemsa stain to observe the RBC morphology under a microscope. Handling methods for whole blood samples include 1) spiking the working solution only, 2) up-and-down mixing after the addition of MeOH, and 3) vortexing for 10 min after the addition of MeOH. The results showed that up-and-down mixing after the addition of the precipitant was sufficient to lyse RBCs in fresh whole blood. The cells in the cryopreserved whole blood which has been cryopreserved for a long time showed a lysis state even after only spiking handling (Figure 2). Therefore, the sample cleanup method in this study was capable of lysing RBCs.

### 3.3 Liquid chromatography–tandem mass spectrometry method validation

#### 3.3.1 Selectivity

There was only negligible interference in all double blank samples at the retention time of SRL. Good selectivity was confirmed between SRL and SRL-d_3_.

#### 3.3.2 Linearity and lower limit of quantification

The MS response was linear across the calibration range for SRL with a correlation coefficient no less than 0.990. The S/N of the LLOQ of SRL was > 5.

#### 3.3.3 Accuracy and precision

The accuracy and precision results are summarized in Table 3. Both intra- and inter-batch accuracy and precision were acceptable (LLOQ QC: RE and RSD are < 20%, and others: RE and RSD are < 15%).

**TABLE 3:** Intra-batch and inter-batch precision and accuracy for SRL in cryopreserved and fresh human whole blood.

Matrix	Intra-batch (*n* = 6)								Inter-batch (*n* = 6 × 3)							
	LLOQ QC		LQC		MQC		HQC		LLOQ QC		LQC		MQC		HQC	
	A	P	A	P	A	P	A	P	A	P	A	P	A	P	A	P
Cryopreserved human whole blood	−2.4	8.0	−0.7	12.8	−2.7	6.8	4.3	7.7	−4.0	8.8	−11.3	10.5	1.3	7.9	5.7	8.5
Fresh human whole blood	11.8	2.9	4.0	6.4	0.0	6.7	3.0	9.0	5.0	13.0	2.0	10.5	0.0	7.3	6.8	7.3

Note: A, accuracy and data are expressed as relative error (RE, %); P, precision, and data are expressed as the relative standard deviation (RSD, %); n, number of replicates; LLQC, 0.500 ng/ml; LQC, 1.50 ng/ml; MQC, 15.0 ng/ml; HQC, 40.0 ng/ml.

#### 3.3.4 Recovery and matrix effect

The extraction recovery, measured at three different concentrations over the whole calibration range (n = 6 for each individual concentration level), was adequate. The post-extraction addition tests show that ion suppression or ion enhancement was not a problem with the present method (Table 4).

**TABLE 4:** Recovery and matrix effect of SRL in cryopreserved and fresh human whole blood.

Nominal conc. (ng/ml)	Recovery (*n* = 6)						IS-normalized matrix factor (*n* = 3 × 6)					
	Cryopreserved human whole blood			Fresh human whole blood			Cryopreserved human whole blood			Fresh human whole blood		
	Mean (%)	RSD (%)	Total RSD (%)	Mean (%)	RSD (%)	Total RSD (%)	Mean ± SD (%)	RSD (%)	Total RSD (%)	Mean ± SD (%)	RSD (%)	Total RSD (%)
1.50	88.6	5.6	4.7	96.7	12.6	9.4	112.2 ± 7.6	6.8	4.2	109.0 ± 3.1	2.8	3.1
15.0	81.0	4.8		80.7	6.4		103.8 ± 4.1	3.9		102.6 ± 3.3	3.2	
40.0	87.2	6.1		93.7	12.1		111.1 ± 8.0	7.2		104.8 ± 3.5	3.3	

Note: RSD, relative standard deviation; total RSD, the RSD for three concentration levels; n, number of replicates.

#### 3.3.5 Stability

The stability of SRL in human whole blood at room temperature, at 4°C in the auto-sampler, and at −80°C for the long term and after five freeze–thaw (−80°C) cycles were acceptable as shown in Table 5.

**TABLE 5:** Stability of SRL in cryopreserved and fresh human whole blood (*n* = 3).

Matrix	Storage conditions		RE (%)	RSD (%)
Cryopreserved human whole blood	Room temperature stability	LQC	−2.0	7.5
	(25°C, 24 h)	HQC	0.5	11.2
	Freeze–thaw stability	LQC	0.0	6.7
	(−80°C, five cycles)	HQC	7.8	3.0
	Autosampler stability	LQC	10.7	12.0
	(4°C, 2 d 17 h)	HQC	7.0	3.3
	Long-term stability	LQC	13.3	6.5
	(−80°C, 31 d)	HQC	0.5	3.0
Fresh human whole blood	Room temperature stability	LQC	13.3	5.3
	(25°C, 24 h)	HQC	9.5	8.7
	Freeze–thaw stability	LQC	−10.0	13.3
	(−80°C, five cycles)	HQC	1.5	9.6
	Autosampler stability	LQC	8.7	12.9
	(4°C, 2 d 17 h)	HQC	3.2	8.0
	Long-term stability	LQC	9.3	0.6
	(−80°C, 31 d)	HQC	3.2	10.4

Note: RE, relative error; RSD, relative standard deviation; LQC, 1.50 ng/ml; HQC, 40.0 ng/ml.

### 3.4 Enzyme multiplied immunoassay technique assay

A calibration curve with a range of 3.00–36.0 ng/ml was automatically obtained from the Viva-E automatic enzyme immunoassay analyzer, while the analyzer has a reportable concentration range of 3.50 ng/ml (based on detection limit and instrument sensitivity) to 30.0 ng/ml. Quantitative results above 30.0 ng/ml can be evaluated by diluting and re-assaying the sample at a higher concentration and multiplying the result by the dilution factor. The concentration was calculated by the formula as shown in Table 6.

**TABLE 6:** EMIT Formula for SRL concentration calculation.

A = a (I) + b (I) * (C – C (I)) *c (I) * (C – C (I)) ^2 + B (I) * (C – C (I)) ^3			
a (0) = −4.80830E-005	b (0) = 0.00000E+000	c (0) = 1.77636E+001	d (0) = −2.08016E-001
a (1) = −6.54051E-005	b (1) = −2.46666E-006	c (1) = 1.77636E+001	d (1) = 9.57422E-002
a (2) = −2.06495E-005	b (2) = −4.74276E-006	c (2) = 1.77636E+001	d (2) = 3.01801E-001
a (3) = 5.99687E-005	b (3) = −5.91977E-006	c (3) = 1.77636E+001	d (3) = 6.39310E-001
a (4) = 6.59947E-006	b (4) = −3.24694E-007	c (4) = 1.77636E+001	d (4) = 1.19176E+000

Inaccuracy = 1.32798E+000.

Our laboratory conducted three concentration levels of QC samples to control inter-day variation. The deviations of QC samples over the period of clinical sample collection and detection were from −13.6% to 14.6%.

### 3.5 Comparison of sirolimus concentrations generated by liquid chromatography–tandem mass spectrometry and by enzyme multiplied immunoassay technique

To the best of our knowledge, this is the first study focusing on the consistency evaluation of SRL concentrations generated by the EMIT and LC-MS/MS. Briefly, 114 blood samples were measured by two methods. SRL concentrations measured by the EMIT and by LC-MS/MS were 11.0 ng/ml (median, range 3.60–41.6 ng/ml) and 7.61 ng/ml (median, range 1.27–34.5 ng/ml), respectively. The median concentration of whole blood SRL determined by the EMIT was higher by 1.45 fold than that by LC-MS/MS.

**FIGURE 3:**
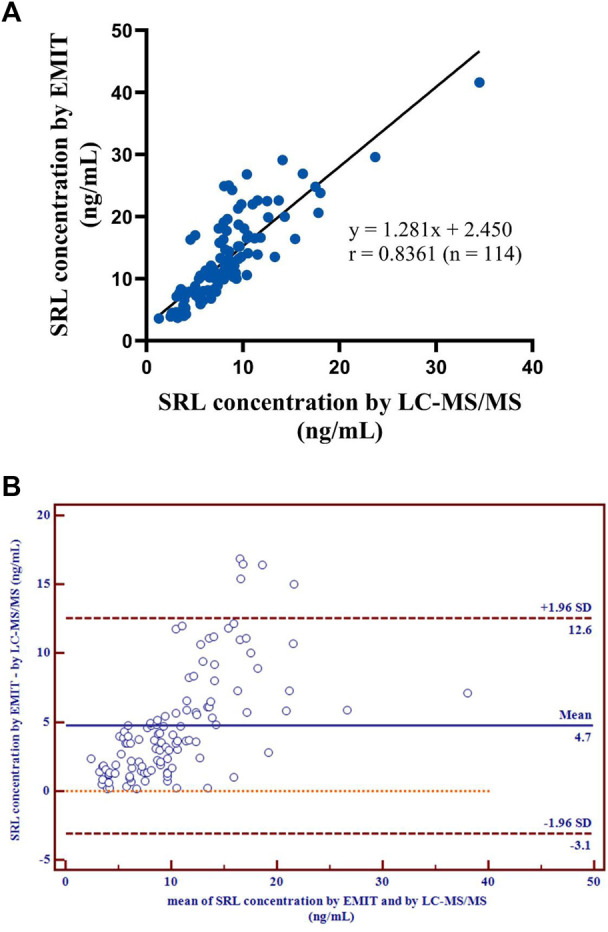
Correlation of the regression curve for LC-MS/MS and EMIT assay (*n* = 114) **(A)**. Differences between mean whole blood SRL concentrations (ng/ml) generated by LC-MS/MS and by EMIT assay expressed as absolute bias (*n* = 114) **(B)**.

Kolmogorov–Smirnov analysis and D’Agostino–Pearson test both revealed that the distribution style of the concentration data obtained from the LC-MS/MS or EMIT method was non-normal distribution. Spearman’s correlation analysis indicated that the data from two assays were significantly correlated (*p* < 0.0001). A regression equation was obtained as follows:

[***EMIT***] = 1.281 × [***LC−MS/MS***] + 2.450

with r = 0.8361 (Figure 3A), which revealed a good correlation between the two methods.

**FIGURE 4:**
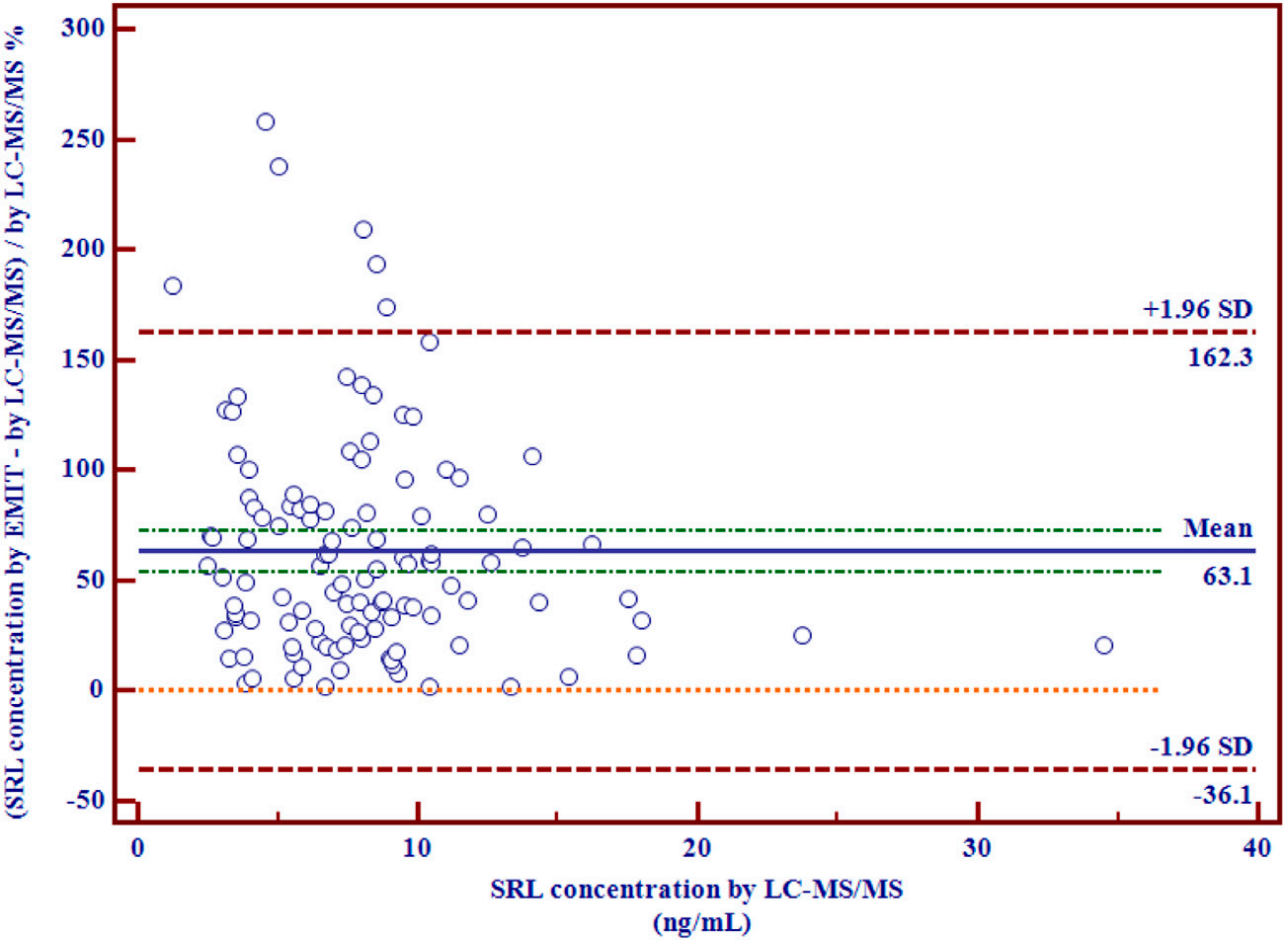
Relative differences between mean whole blood SRL concentrations (ng/ml) generated by LC-MS/MS and by the EMIT assay expressed in percentage (*n* = 114).

**FIGURE 5:**
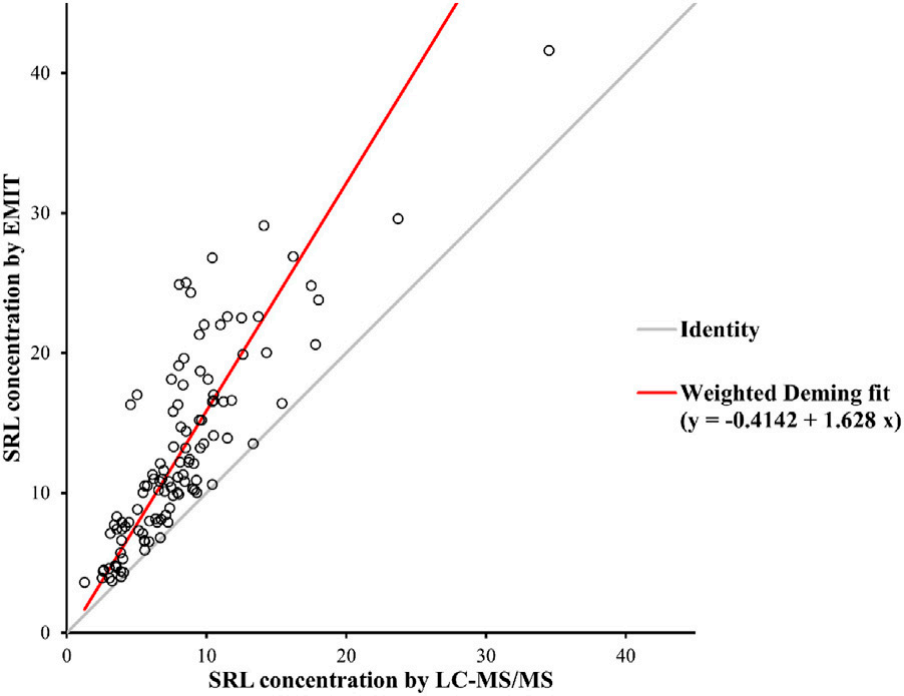
Weighted Deming Plot between SRL concentrations generated by the EMIT and LC-MS/MS methods.

There were disparities between the SRL concentrations generated by the EMIT and by LC-MS/MS plotted against the mean level determined by two methods (Figure 3B). The levels of the whole blood SRL measured by the EMIT were higher than those determined by LC-MS/MS [positive bias: 4.7 ng/ml; 95% CI: (−3.1, 12.6)]. The Bland–Altman difference plot in Figure 4 shows the relative difference calculated by [(EMIT)–(LC-MS/MS)/(LC-MS/MS)], plotted against the LC-MS/MS results. There was a mean positive bias of 63.1% [95% CI: (−36.1, 162.3)] compared with the LC-MS/MS assay. Overall, the Bland–Altman difference plots suggested that the EMIT systematically overestimated SRL levels in whole blood compared to LC-MS/MS data. In addition, the data comparison was also performed by Weighted Deming regression. The Deming plot also revealed a mean positive bias for the EMIT (Figure 5).

The EMIT is easily operated but has a number of shortcomings such as high cost per sample, low specificity, and falsely elevated concentrations (Mika and Stepnowski, 2016). LC-MS/MS is currently used as a gold standard assay for measuring the concentration of SRL because it is known for negligible interference (Lee, Kim et al., 2019). In the current study, the whole blood concentration of SRL from the EMIT was higher than that generated by the LC-MS/MS method. A mean overestimation of about 63.1% was observed. Similar results were reported in other comparison studies performed to investigate the difference between the immunoassay and LC-MS/MS. Fillee, Mourad et al. (2005) found a global overestimation of about 15% by microparticle enzyme immunoassay (MEIA) compared with LC-MS/MS. The mean MEIA bias was found to be 11.5% compared with LC-MS/MS in a correlation study by Vicente, Smith et al. (2006), and Salm, Taylor et al. (2000) revealed a mean overestimation of 42.5% by MEIA compared with LC-MS/MS. The difference between these MEIA studies and the present study may be due to the different determination principles of MEIA and the EMIT.

In general, immunoassay results were overestimated compared to LC-MS/MS (Lee, Kim et al., 2019). LC-MS/MS has excellent reproducibility and low interference, suggesting that it is an unlikely source of overestimation. The most likely explanation for the bias would be non-specific binding to antibodies, known as cross-reactivity. Immunoassay cross-reactivity has been demonstrated between SRL and its 41-O-desmethyl (86–127%) and hydroxy (44–50%) metabolites (Jones, Saadat-Lajevard et al., 2000). In addition, SRL and its major metabolites have significant cross-reactivity with everolimus (Baldelli, Crippa et al., 2006; Bouzas and Tutor, 2007; Khoschsorur, Fruehwirth et al., 2007), although they are unlikely to be administered simultaneously to patients. Moreover, the matrix effect can also significantly compromise the performance of immunoassays (Sallustio, Noll et al., 2011). Additionally, deviations may also be caused by inaccurate calibration before measurement and hematocrit (Sallustio, Noll et al., 2011; Sturgeon and Viljoen, 2011). Therefore, all of these potential causes likely act in a synergic way, and the final effect observed is hardly due to the cross-reactivity.

One more question needs to be further considered. Early studies suggested a whole blood SRL therapeutic window of 5–15 ng/ml or 6–12 ng/ml (in combination with tacrolimus) using MEIA as the detection method (MacDonald, Scarola et al., 2000; McAlister, Gao et al., 2000). Another report in the same year showed that a trough concentration window of 5–15 ng/ml measured by an HPLC instrument combined with an ultraviolet detector (HPLC-UV) could be regarded as the putative target for dose tailoring (Kahan, Napoli et al., 2000). Furthermore, the therapeutic window recommended for trough SRL concentration in patients on triple therapy with cyclosporine, corticosteroids, and SRL was 4–12 ng/ml, determined by HPLC-UV or LC-MS/MS (Oellerich, Armstrong et al., 2004). These reports revealed that the detection methods had no influence on the target range definition for blood SRL monitoring. Our study found a good correlation between the EMIT and LC-MS/MS, indicating that switching between the two methods was feasible. However, when switching from the EMIT to an alternative LC-MS/MS method, clinical TDM laboratories need to explain the results to clinicians and patients that the decrease in concentration results was due to an overestimation of the previous EMIT. More emphasis should be simultaneously placed on efficacy and safety rather than just concentration data.

## 4 Conclusion

This study compared the whole blood SRL concentrations generated by a routine EMIT and by a newly validated LC-MS/MS assay using a number of whole blood samples from children with vascular anomalies. In summary, there was a close correlation between the two methods, but EMIT assay significantly overestimated SRL concentrations by 63.1% compared with the LC-MS/MS method. Switching from the EMIT to the LC-MS/MS technique for routine TDM of SRL deserves great concern. Moreover, the results generated by LC-MS/MS are closer to the true values; therefore, necessary re-evaluation for the target therapeutic reference range may be required when methods are switched within the same clinical laboratory or results are compared between different laboratories.

## Data Availability

The original contributions presented in the study are included in the article/Supplementary Material; further inquiries can be directed to the corresponding authors.
